# Novel wireless electroencephalography system with a minimal preparation time for use in emergencies and prehospital care

**DOI:** 10.1186/1475-925X-13-60

**Published:** 2014-05-08

**Authors:** Andrei Jakab, Antti Kulkas, Timo Salpavaara, Pasi Kauppinen, Jarmo Verho, Hannu Heikkilä, Ville Jäntti

**Affiliations:** 1 Department of Electronics and Communications Engineering, Tampere University of Technology, Korkeakoulunkatu 3, FI-33720 Tampere, Finland; 2 Department of Clinical Neurophysiology, Seinäjoki Central Hospital, Hanneksenrinne 7, FI-60220 Seinäjoki, Finland; 3 Department of Automation Science and Engineering, Tampere University of Technology, Korkeakoulunkatu 3, FI-33720 Tampere, Finland

**Keywords:** Electroencephalography (EEG), Wireless EEG recorder, Prehospital care, Ambulance recordings, Altered mental state

## Abstract

**Background:**

Although clinical applications such as emergency medicine and prehospital care could benefit from a fast-mounting electroencephalography (EEG) recording system, the lack of specifically designed equipment restricts the use of EEG in these environments.

**Methods:**

This paper describes the design and testing of a six-channel emergency EEG (emEEG) system with a rapid preparation time intended for use in emergency medicine and prehospital care. The novel system comprises a quick-application cap, a device for recording and transmitting the EEG wirelessly to a computer, and custom software for displaying and streaming the data in real-time to a hospital. Bench testing was conducted, as well as healthy volunteer and patient measurements in three different environments: a hospital EEG laboratory, an intensive care unit, and an ambulance. The EEG data was evaluated by two experienced clinical neurophysiologists and compared with recordings from a commercial system.

**Results:**

The bench tests demonstrated that the emEEG system's performance is comparable to that of a commercial system while the healthy volunteer and patient measurements confirmed that the system can be applied quickly and that it records quality EEG data in a variety of environments. Furthermore, the recorded data was judged to be of diagnostic quality by two experienced clinical neurophysiologists.

**Conclusions:**

In the future, the emEEG system may be used to record high-quality EEG data in emergency medicine and during ambulance transportation. Its use could lead to a faster diagnostic, a more accurate treatment, and a shorter recovery time for patients with neurological brain disorders.

## Background

The World Health Organization classifies neurological disorders as a great threat to public health since these disorders and their sequelae are estimated to affect approximately one billion people worldwide [1]. One of the most commonly used tests for the evaluation of neurologic disorders is the electroencephalogram (EEG) [2]. It is invaluable since: it can help a physician distinguish among different types of unconsciousness and comas; it is the only method for continuously monitoring cerebral function over prolonged periods of time; and, it is the only specific test for epileptic disorders [3,4]. Additional acute indications for the EEG include the investigation of strokes, head trauma, and intracranial haemorrhages [5-7]. During the last 10 years, the EEG has become the first examination of unconscious patients in the emergency rooms of several hospitals [3].

Traditional EEG recording methods are relatively time-consuming, require specialized personnel, and the recording systems are cumbersome. Hence, they are not optimized for use in emergency medicine (EM), and especially in prehospital care, i.e., ambulances and other specialized environments, where the challenge is to diagnose neurological disorders quickly so that specific treatment can be started as early as possible. It has been shown that patients with altered mental states (AMS), in particular, would benefit from EEG recording done early in the diagnosis process [8,9].

In recent years, a number of ambulatory EEG systems have been developed [10-15]. Nevertheless, since most of these have an intended application area other than emergency medicine, e.g., brain-computer interface, home healthcare, epilepsy monitoring, their size, weight, carrying system, electrode harness, and/or need to be assembled prior to use makes them difficult to employ in EM and prehospital care. Recently there have also been efforts to develop systems specialised for EM [16].

One of the important design decisions in the development of an EM EEG system is the choice of electrodes. Currently wet-gel Ag/AgCl electrodes are typically used in clinical EEG applications since they offer excellent signal quality and are widely available commercially. However, these types of electrodes typically require some kind of skin preparation before they can be reliably applied [17]. An interesting alternative to wet electrodes are dry electrodes, which do not require conductive electrode paste or skin preparation [18,19]. Nevertheless, dry electrodes still suffer from signal quality limitations, which are due to their sensitivity to noise and motion artefacts [17]. Wet electrodes typically have adhesive material to keep them relatively immobile as well as conductive gel to lower the skin impedance and protect the electrode against motion artefacts. Currently dry electrodes are not widely available for clinical use in EEG recordings, which further limits their usability [17].

The aim of the current work was to develop and test a novel system for performing EEG recordings in EM and in ambulances, while patients are being transferred to the hospital. This is a vital issue because even though EEG is an important tool for assessing brain function, it is not yet clinically available in prehospital care partly due to the lack of specifically designed recording systems for this challenging environment. High-quality EEG data in EM and during ambulance transportation could lead to a faster diagnostic, more accurate treatment, and shorter recovery of patients with neurological brain disorders.

An ideal recording system for these environments should be portable, small, robust, and easy-to-use so that even caregivers that are not specifically trained in EEG recordings can operate it. Also, the time required for patient preparation should be minimized and the data should be made available to a hospital-based interpreter in real-time. Finally, the system's signal quality should be comparable to that of traditional recorders.

## Methods

### System overview

**Figure 1:**
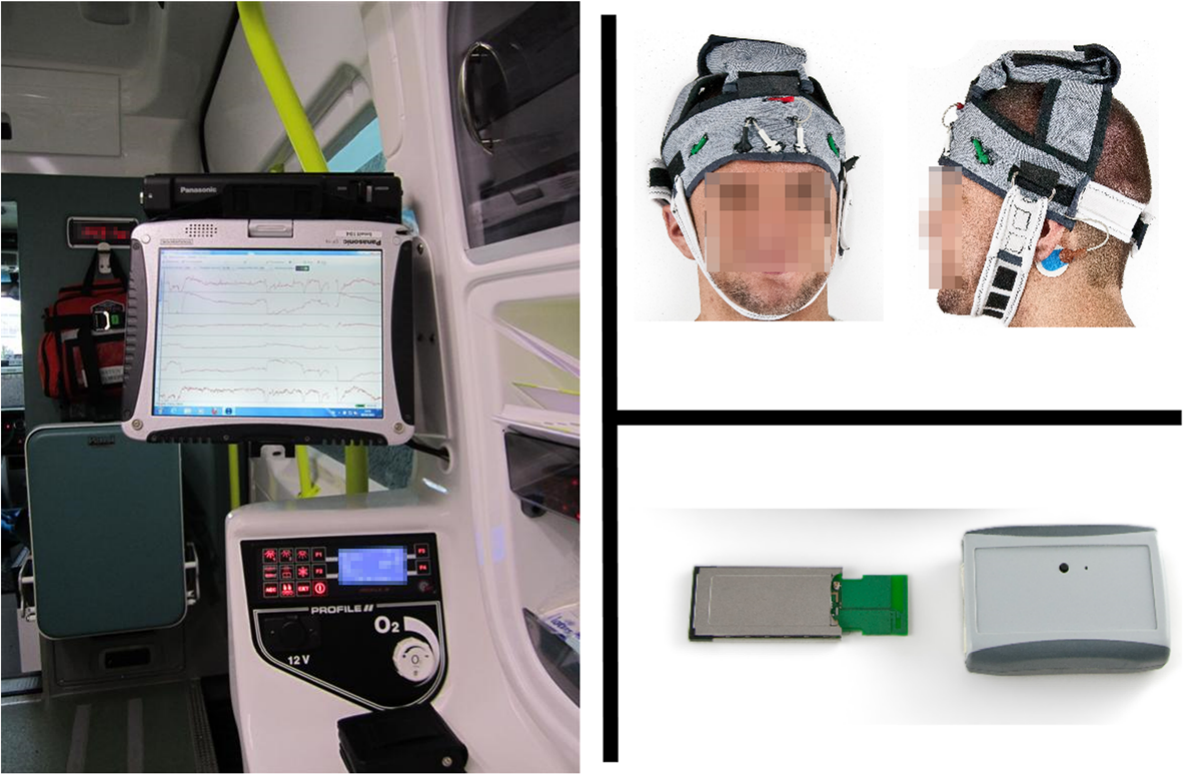
**The emEEG system.** It consists of recording software (left), quick-application electrode cap (top-right), and wireless EEG recorder (bottom-right).

The developed 6-channel emergency EEG system (emEEG), which is shown in Figure 1, is comprised of three separate entities: a quick-application EEG cap into which the measurement electrodes are embedded; a wireless EEG (WEEG) recorder that measures the EEG and transmits it wirelessly to a personal computer; and recording software, which displays and stores the data, allows it to be annotated, and streams it in real-time to a remote server where it can be analyzed by a clinician.

#### *Wireless EEG recorder*

**Figure 2:**
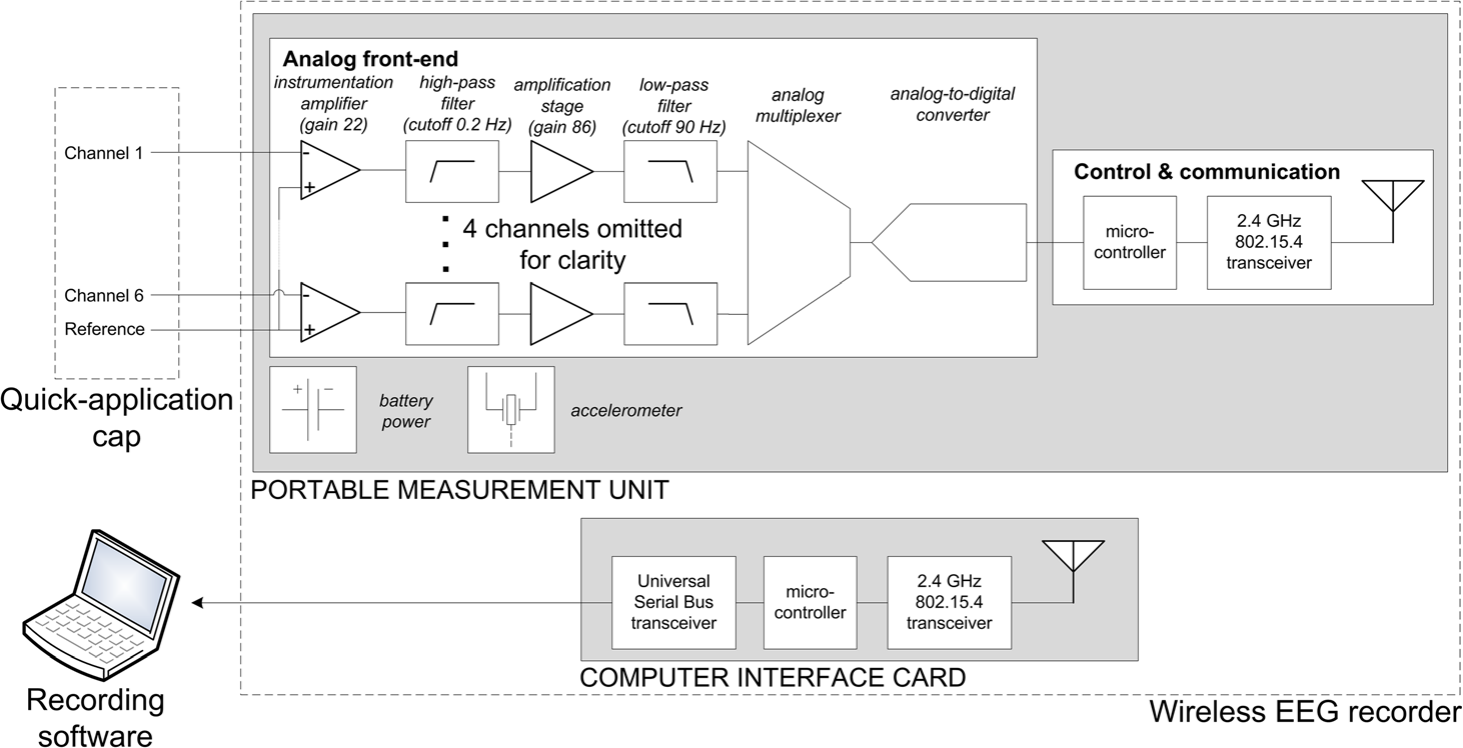
**System diagram depicting the major components of the six channel emEEG system.** For clarity, only two of the six measurement channels are shown.

The WEEG recorder consists of a portable measurement unit (PMU; 9.3 × 6 × 2.5 cm, 75 g) and a computer interface card (CIC; 7.5 × 3.4 × 5 cm, 7.3 g), the latter of which is located within the measurement laptop computer. Table 1 summarizes the specifications for the WEEG and a commercially available NicoletOne V32 (Natus Medical Inc, San Carlos, USA) amplifier. Block diagrams of the PMU and CIC are shown in Figure 2, with each major part being described in the subsequent paragraphs.

**Table 1:** Summary of the characteristics of the developed emEEG device and of a commercial device

**Device**	**emEEG**	**NicoletOne V32**
**Number of EEG channels**	6	32
**ADC resolution (bits)**	16	16
**Voltage resolution (μV)**	0.02	0.15
**Maximum sampling rate (Hz)**	1000	2000
**Bandwidth (Hz)**	0.2 – 90	0.05 – 500
**CMRR (dB)**	80	>115
**Input impedance (MΩ)**	≈1000	>100
**Acceleration information**	Three axes	none

The design of the PMU’s analog front-end follows that of a typical bioamplifier [20]. Even though it has been omitted from Figure 2 for clarity purposes, the input of each channel possesses a voltage-limiting circuit, which prevents the high-voltage transients induced by defibrillators from damaging the PMU. The EEG signal is pre-amplified by means of an INA2126 (Texas Instruments, Dallas, USA) instrumentation amplifier (IA). Due to its low voltage noise (45 nV/√Hz), high input impedance (>1 GΩ), high common-mode rejection (≥ 80 dB) this IA is ideal for amplifying the small-amplitude EEG signal. A signal conditioning section follows, in which the signal's DC offset is removed by means of a high-pass (HP) filter (f_c_ ≈ 0.2 Hz, 1st order Butterworth), prior to the signal being further amplified by a gain stage. After low-pass (LP) filtering (f_c_ ≈ 90 Hz, 2nd order Bessel), the EEG signal is digitized by a 16-bit analog-to-digital converter (ADC). Sampling frequencies in the 200 – 1000 Hz range can be used to digitize the signal.

Movements of the patient's head can cause significant artefacts in the recorded EEG signal. Therefore, an accelerometer is included in the PMU in order to allow the caregiver to ascertain whether an abnormal EEG feature is caused by the underlying neurophysiology or by movement. The accelerometer employed in this design is the ADXL330 (Analog Devices, Norwood, USA). It can measure acceleration in three directions, has a typical range of ± 3.6 g, and consumes only 1.2 mW.

In the control and communication block the main component is an ATmega128L microcontroller (Atmel Corporation, San Jose, USA), which features an 8-bit high-performance, low-power architecture. The principal task of the microcontroller is to group the EEG samples together with the 10-bit acceleration data and to transfer the resulting packets to the CIC through a custom wireless protocol, which is built on top of the IEEE 802.15.4 standard's physical and media access control layers. This communication method was chosen due to its low power consumption, ease-of-use, and adequate performance. A CC2420 transceiver chip (Texas Instruments, Dallas, USA) handles the wireless communication and operates in the 2.4 GHz band, which is reserved internationally for industrial, scientific and medical purposes. Previous studies have shown that transmissions at this frequency do not interfere with common medical devices [21,22].

The computer interface card transmits the data packets received from the PMU to the recording software and vice versa. It features an ExpressCard interface and a U.FL miniature coaxial connector (Hirose Electric Group, Tokyo, Japan) for interfacing with one of the laptop's 2.4 GHz 802.11 wireless local area network (WLAN) antennas. Therefore, any modern laptop with an ExpressCard interface as well as two or more 2.4 GHz 802.11 antennas can be employed to record EEG data using the emEEG system.

While the CIC draws power directly from the measurement computer through the ExpressCard interface, the PMU is powered by three AAA batteries. Using Duracell Coppertop MN2400 alkaline batteries (The Procter & Gamble Company, Bethel, USA), a sampling frequency of 200 Hz, and with the CIC and PMU adjacent to each other, it was possible to operate the recorder continuously for approximately 23 hours.

#### *Recording software*

Custom software controls the operation of the WEEG recorder and displays the measured EEG and acceleration data. It also allows the data to be annotated using pre-defined and custom annotations. Furthermore, the displayed EEG data can be LP filtered simply by selecting the desired cut-off frequency from the toolbar. Raw EEG and acceleration data are stored side-by-side in an EDF+ file, which can be opened by the majority of EEG analysis software. While a measurement is in progress, the recorded data is streamed to a remote server through any internet connection that the measurement computer may possess, e.g., mobile broadband, WLAN, satellite. This could allow a clinician to ascertain a patient's condition before they even reach a medical center.

#### *Quick-application cap*

The cap was constructed with polyester-based materials that can be easily sterilized, either by washing or by wiping with detergent. It was designed so that it could potentially be applied to a patient that has suffered a neck injury and who is lying in the supine position on a flat surface, albeit before the fitting of a cervical collar or head immobilization device. The WEEG recorder is housed within one of the structural bands on the apex of the head. Conductive carbon fibres embedded within the bands' material diminish the possibility of electrostatic discharges, which could damage the measurement electronics.

Six electrode locations were selected from the international 10–20 system by means of finite element modeling and lead field theory. The number of measurement channels was set to six due to the limited bandwidth of the wireless link and to ensure a quick application of the system. Only sub-hairline locations were considered so as to allow the pre-gelled EEG electrodes described subsequently to be used. An additional constraint imposed on the analysis was for the locations to be symmetric along the sagittal plane so as to allow the detection of neurological asymmetries. Wendel created a realistic four-shell head model with which the sensitivity distributions of six possible electrode configurations matching the aforementioned requirements were compared [23]. The configuration comprising the following locations was shown to have the broadest sensitivity distribution: P9, F7, Fp1, Fp2, F8, and P10. An additional location is used as the reference (Fpz) and another as the electronic 'ground' (Fz).

Disposable wet-gel Ag/AgCl snap electrodes were used in this study, the suitability of which for EEG recordings has been demonstrated in literature [24-28]. Compared to traditional EEG electrodes, e.g., cup electrodes, these wet-gel snap electrodes can be applied very quickly since the electrolyte gel and the adhesive do not have to be added manually. Furthermore, extensive skin preparation is not required since it has been shown that excellent electrode impedances can be obtained with only routine skin defatting [26,28].

### Testing procedure

The emEEG system's suitability for testing in a clinical environment was ascertained through benchtop tests, which investigated the WEEG recorder's characteristics, as well as healthy volunteer and patient measurements that established the system's accuracy by performing data comparison with a commercial EEG system. The study protocol was reviewed by the regional ethical committee of the Tampere University Hospital, which issued a positive statement regarding healthy volunteer and patient measurements. All participants gave their written consent to take part in the study.

#### *Benchtop testing*

The frequency responses of the WEEG recorder's six channels were measured using the standard frequency sweep method. The common-mode rejection ratio (CMRR) of a bioamplifier is defined as the ratio of the differential gain A_D_ over the common-mode gain A_CM_ and is an indication of the amplifier's capability of rejecting common-mode signals. Its value is expressed in decibels (dB):

(1) $$\mathrm{CMRR}=20\log_{10}\left(\frac{A_{D}}{A_{\mathrm{CM}}}\right)$$

The A_CM_ of each channel was determined by feeding a 50 Hz sinusoidal signal to the six input channels as well as to the reference channel and then measuring the voltage at the output of the IA stage. This frequency was chosen because 50/60 Hz mains interference is a common source of common-mode noise in EEG recordings [14]. A channel's A_D_ was obtained by measuring the IA's output when a sinusoid generated by a XC90-III medical calibrator (Oxford Instruments, Abingdon, England) was inputted differentially. Subsequently, the channels' CMRR was calculated by means of Equation 1.

The noise level was measured in a laboratory environment by connecting together the PMU's six input channels, the reference channel, and the bias connection. With the inputs short-circuited in this way, the output of the PMU is equal to its internal noise. A one-minute recording was performed and the root mean square (RMS) amplitude of the signal measured by each channel was calculated using MATLAB (The MathWorks, Natick, USA). The RMS noise level of each channel at the input of the ADC was calculated from the measured signals after the DC offset was removed with a linear-phase finite impulse response (FIR) HP filter with a cut-off frequency of 0.2 Hz. The noise at the input of the adapter was estimated by dividing the noise at the ADC input by each channel's measured A_D_.

#### *Parallel recordings*

In order to verify the accuracy of the emEEG system, the EEG signals recorded with it were compared to reference signals acquired with a NicoletOne V32 commercial EEG measurement system (Natus Medical Inc, San Carlos, USA). The NicoletOne V32 was chosen as the reference system since it is in clinical use at the Seinäjoki Central Hospital (Seinäjoki, Finland). The spontaneous EEG of two healthy male volunteers was measured simultaneously using the two systems, first in an examination room at the Department of Clinical Neurophysiology, and then in the intensive care unit (ICU) of the Seinäjoki Central Hospital. Zipprep electrodes (Aspect Medical Systems, Inc, Washington USA) were used in these recordings and the measurements were preceded by routine defatting of the electrode locations using alcohol pads. While lying in the supine position, the subject was first asked to blink their eyes for 10 seconds and then keep them closed for three minutes. This procedure was repeated three times during the approximately 10-minute-long recordings. The leads of the quick-application cap were connected to the two systems so as to be able to measure the subject's EEG simultaneously using both systems and from the same locations. Prior to the start of the measurements, all channels were checked to ensure that low and similar impedances were present (< 5 kΩ).

Because the recordings were not started at exactly the same time and since the sampling rates of the two systems were not completely identical, the emEEG signals were resampled to the time points of the commercial system by means of a simple algorithm. First, the bandwidth of both signals was reduced to the 0.5 – 35 Hz range, which is typically used in EM, by means of HP and LP linear-phase FIR filters. In order to ensure a flat pass-band, both filters were designed by the windowing technique with the Kaiser window. Second, the time offset between the two signals was determined at four different locations evenly distributed over the recording. Identical features present in both signals were identified and the amount of samples separating them was calculated. These offsets were subsequently used to compute the coefficients of the first-degree polynomial that models the time offset between the two signals. Thirdly, the data recorded with the emEEG system was mapped to the timeline of the commercial data by means of the derived polynomial. Finally, the emEEG data was linearly interpolated at the time points of the commercial instrument’s data.

#### *Patient measurements in hospital EEG laboratory and ICU*

Patient measurements were performed at the Department of Clinical Neurophysiology and ICU of the Seinäjoki Central Hospital. Every EEG recording made with the emEEG system was preceded with a standard clinical EEG recording performed with a commercial system. Zipprep electrodes were used in these recordings and the measurements were preceded by routine defatting of the electrode locations using alcohol pads. The emEEG recordings were approximately 20 minutes in length. Two experienced clinical neurophysiologists independently evaluated the technical quality of the emEEG recordings and whether the signal quality was sufficient for clinical interpretation. They also gave their statement on the patients’ neurological condition blindfolded, i.e., without prior knowledge of the patients and their condition. The accelerometer data was visible to the interpreter during analysis of the emEEG recordings to support the determination of whether a particular signal feature is a movement artefact or not. According to the study protocol, a three-level grading system was used for the evaluation: normal, abnormal, and highly abnormal. The clinicians also indicated whether status epilepticus was suspected. The results were then compared with the statements made based on the standard clinical EEG recordings.

### Preliminary ambulance recordings

After the clinical testing, the developed emEEG system was installed in an ambulance. The laptop computer was docked to the ambulance (Figure 1) and EEG recordings were performed for two male healthy volunteers with Norotrode electrodes (Myotronics Inc., Washington USA) while the vehicle was moving. The measurements were preceded by routine defatting of the electrode locations using alcohol pads. During the measurements, the ambulance drove through the city area of Seinäjoki as well on the nearby highway. An experienced clinical neurophysiologist then evaluated the technical quality of the EEG recordings.

## Results

### Laboratory tests

Through the frequency response measurements the system's high-pass and low-pass -3 dB points were determined to be 0.19 and 90 Hz, respectively. Also, the gain in the flat region of the passband was found to be approximately 1817 V/V (65.19 dB). The frequency responses of the six channels were nearly identical, with a maximum difference of 0.34 dB over the 0.19 – 90 Hz range.

The average CMRR was found to be 83 dB with a standard deviation (SD) of 0.74 dB (see Table 2). Also, the CMRR of all channels exceeds the 80 dB threshold recommended by the ACNS guidelines [29].

**Table 2:** Common mode rejection and root mean square noise level of the emEEG system’s six channels

**Channel**	**CMRR (dB)**	**Channel input noise (μV**_**RMS**_**)**
**P10**	83	0.40
**F8**	82	0.40
**Fp1**	84	0.40
**Fp2**	82	0.42
**F7**	82	0.41
**P9**	82	0.40
**Average**	83	0.41

The CMRR was computed at 50 Hz and the RMS noise was calculated over the 0.2 – 90 Hz frequency range.

All six channels exhibited excellent noise properties over the 0.2 – 90 Hz frequency range (see Table 2). Although this measurement technique does not take into account the Johnson-Nyquist noise generated by the electrode impedance and the noise generated by the IA's input bias current, under typical recording conditions these values will be small and therefore their contribution to the overall noise present in the recorded signal was deemed negligible. The average input noise was found to be 0.41 μV_RMS_ with a SD of 7.6 nV_RMS_.

### Parallel recordings

**Figure 3:**
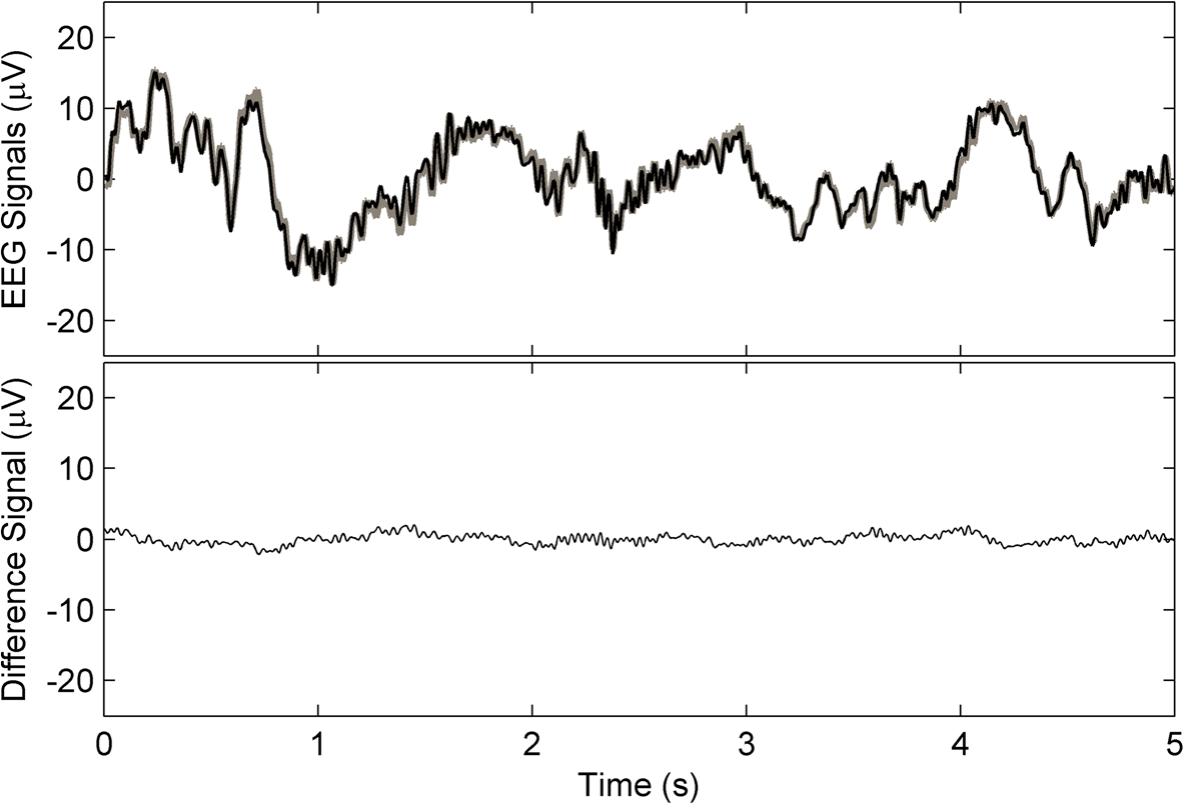
**Comparison of two signals recorded in parallel with the emEEG and a NicoletOne V32 system.** At the top is the comparison of a 5 seconds window of the EEG signals measured with the two systems from subject 1 in the ICU of the Seinäjoki Central Hospital (emEEG signal in black and NicoletOne V32 signal in gray). At the bottom is the computational difference of the two signals (mean difference is 0.59 μV and standard deviation is 0.44 μV).

Since the preparation needed with the novel EEG system is minimal, the recordings were started in less than three minutes. A five second window of the comparison results is shown in Figure 3. The upper part of the figure depicts the reference signal together with the signal measured by the emEEG system while the lower part presents the minute difference between the two signals. The mean differences between the two signals for the four recordings are presented in Table 3. Considering that the amplitude of non-pathological scalp EEG is usually in the 10 – 100 μV range, the comparatively small mean difference proves that the EEG measured with the emEEG system is very similar to that recorded with the commercial system.

**Table 3:** Comparison of data recorded in parallel with the emEEG system and a NicoletOne V32 system

**Channel**	**Examination room**		**ICU**		**Average**
	**Subject 1**	**Subject 2**	**Subject 1**	**Subject 2**	
**P9**	1.3 ± 1.6	1.3 ± 1.6	1.0 ± 1.4	1.4 ± 1.9	1.3 ± 1.6
**F7**	1.1 ± 1.3	0.9 ± 1.0	0.8 ± 0.8	0.9 ± 0.9	0.9 ± 1.0
**Fp1**	1.2 ± 1.5	0.9 ± 1.2	0.7 ± 1.0	1.1 ± 1.9	1.0 ± 1.4
**Fp2**	1.0 ± 1.1	0.7 ± 0.8	0.7 ± 0.9	0.7 ± 1.8	0.8 ± 1.1
**F8**	1.3 ± 1.7	0.8 ± 1.1	0.7 ± 0.9	1.0 ± 1.0	0.9 ± 1.2
**P10**	1.2 ± 1.5	1.2 ± 1.5	0.9 ± 1.2	1.2 ± 1.7	1.1 ± 1.5
**Average**	1.2 ± 1.4	1.0 ± 1.2	0.8 ± 1.0	1.1 ± 1.6	1.0 ± 1.3

The table displays the mean difference ± SD of the absolute difference signal computed from the EEGs recorded simultaneously with both systems (units: μV).

### Patient measurements in hospital EEG laboratory and ICU

The patient recordings performed at the Department of Clinical Neurophysiology and at the ICU were also started in less than three minutes. The results are presented in Table 4. In all four cases, the technical quality of the recordings was rated to be sufficient for diagnostic purposes by both of the experienced clinical neurophysiologist and the diagnostic comparison showed consistency with the commercial system.

**Table 4:** Results of the patient measurements

**Patient**	**1**	**2**	**3**	**4**	**5**
**Age**	23	25	55	58	75
**Gender**	Female	Male	Female	Male	Female
**Recording location**	Clin.Depart.	Clin.Depart.	ICU	ICU	Clin.Depart.
**General estimation (clinical EEG)**	Normal	Normal	Highly Abnormal	Highly Abnormal	Highly Abnormal
**Status Epilepticus**	No	No	No	No	Yes
**Experienced clinical neurophysiologist 1**					
**General estimation (emEEG)**	Normal	Normal	Normal	Highly Abnormal	Highly Abnormal
**Status Epilepticus**	No	No	No	No	Yes
**Experienced clinical neurophysiologist 2**					
**General estimation (emEEG)**	Normal	Normal	Normal	Highly Abnormal	Highly Abnormal
**Status Epilepticus**	No	No	No	No	Yes

The results from the patient measurements were evaluated by two experienced clinical neurophysiologists. Three grades were used in the general estimation, normal, abnormal and highly abnormal based on the clinical practice. The recording location at the Department of Clinical Neurophysiology is denoted as Clin.Depart. and the intensive care unit as ICU.

### Preliminary ambulance recordings

The system performed in the ambulance environment as intended. The recorded EEG data from the healthy volunteers was streamed in real-time to the Department of Clinical Neurophysiology at the Seinäjoki Central Hospital and the technical quality of the EEG recordings was rated to be good and sufficient for diagnostic purposes by an experienced clinical neurophysiologist.

## Discussion

The results of the laboratory test and of the healthy volunteer and patient measurements show that the emEEG system performs well in a variety of environments. Due to its high CMRR, low internal noise, and sufficient bandwidth [26,27], the implemented system records high-quality EEG signals. Furthermore, its extended operating time ensures that the system can be used for long-term recordings. Compared to traditional portable recorders, the system is smaller and requires only a brief training to use. Also, unlike existing portable recorders, the emEEG system features a quick-application cap with which the recording can be started quickly and without a lengthy preparation. Qualitative testing has shown that depending on the patient's head size and hair length, the cap can be applied in two to three minutes, with the slowest part being the peeling of the plastic electrode covers. This time would most likely be longer for patients with long hair since the tightening of the attachment straps would be more challenging. One final advantage of the developed emEEG system is its integrated accelerometer, which can be used to assess the movements of the patients and to evaluate resulting movement artefacts in the EEG signals.

As it can be observed from Figure 3 and Table 3, the EEG measured with the emEEG system is very similar to the EEG recorded with the commercial system. Over the four recordings, the average difference between the two systems amounts to 1.0 μV, with a SD of 1.3 μV. Two factors mainly contribute to the difference between the signals recorded with the two systems. First, a small amount of internal noise (≈ 0.41 μV_RMS_) is added to the recorded signal. Second, although it was assumed that the frequency response of both systems is linear in the 0.5 – 35 Hz range, it is likely that the pass band of each system is not completely flat. Whereas the non-uniform frequency responses generate small variances throughout the recording, their effect is emphasized by the large peaks of the ECG artefacts present in the EEG of channels P9 and P10. Hence, the data recorded by these channels exhibits a slightly larger mean difference.

Based on Table 3, since the EEG from the examination room and that from the ICU exhibits similar characteristics, neither of the two environments seems to negatively affect the quality of the recorded data. However, it is acknowledged that further measurements need to be carried out in the ICU in order to determine whether other electrical medical devices, e.g., ECG monitors, catheters, to which a patient may be attached, could interfere with the recording.

Whereas all 21 electrodes of the 10–20 system are used in traditional EEG studies, the emEEG system features six electrodes. This was due to the technical characteristics of the wireless link and also due to the effort to make the system as easy-to-use as possible. Although current clinical guidelines recognize that a smaller number of channels can be appropriate under special circumstances, the data cannot be considered to give a comprehensive view of the patient's neurological status [30]. An even distribution of the six electrodes over the cerebral hemispheres, including hairy regions, could enhance the diagnostics but would at the same time compromise the ease-of-use of the system. Applying electrodes in hairy regions typically requires more preparation to ensure good contact and stability, which leads to increased set-up times. Therefore subhairline electrode locations were chosen in the current work. Nevertheless, with the symmetric disposition of the electrodes, the physician should be able to detect asymmetries, suggesting focal or asymmetric damage. Also, generalized pathologies, e.g., slowing, epileptic discharges, should be readily visible. Subhairline electrode sets with limited amount of electrodes have been demonstrated to be accurate in non-convulsive status epilepticus and seizure detection [31,32]. This conclusion is also supported by the results of the current patient measurements, which showed comparable results with commercial EEG systems.

In patient 3, the frontal EEG consisted mostly of very low amplitude, slow waves, with some beta activity. As the analysis was being carried out blind and since the lack of normal posterior physiological rhythms was not visible in the emEEG recording, the slow activity was thought to be of a non-pathological nature, e.g., drowsiness, sweating. More information regarding the state of the patient might’ve led to a different diagnosis. Furthermore, it must be noted that the emEEG recordings were not made simultaneously with the commercial system so the temporal difference might also influence the interpretation of the EEG data. It is also acknowledged that additional channels would enhance the diagnostics; on the other hand, increasing the number of channels would probably lengthen the setup time and also compromise the system’s applicability and usability in demanding recording situations, e.g., during ambulance transport.

Two types of proprietary wet-gel electrodes were used in this work because they can be applied very quickly and do not necessitate a long preparation of the skin in order to obtain low impedance. It has been shown that such electrodes can be used as EEG electrodes in the assessment of depth of anesthesia [24-26,28]. Also, to the best of our knowledge, no other snap electrodes intended for EEG measurements were available at the time of the study. Zipprep electrodes were used in the hospital testing of the developed system. Due to the limited availability of the Zipprep electrodes, Norotrode electrodes were used for the ambulance testing. The Norotrode electrodes performed well during the measurements and due to their lower cost and better commercial availability, they offer a good alternative to be used in future measurements.

Wet-gel electrodes do have shortcomings as they cannot be applied on hair and the electrode gel can dry out within a few hours of application. It follows that such electrodes can only be used for short-term recordings. One recently-developed alternative could be an EEG monitoring set consisting of 12 hydro-gel coated electrodes [33]. Dry active electrodes could prove to be another alternative solution. Some of these consist of metallic pins that can go through the patient's hair to make contact with the scalp [34,35]. Therefore, dry active electrodes are certainly an interesting alternative to be considered in EEG recordings [19], especially in the hairy regions where wet gel electrodes cannot be easily applied [18]. Also, since dry electrodes do not require electrolyte gel, they can be applied on the patient very quickly. Nevertheless, it must be acknowledged that dry electrodes still need further research in order to overcome the limitations that prevent their widespread use for clinical EEG recordings [17].

To further enhance the performance of the developed emEEG system, future improvements will be focused on the electrode cap design to enable a more even distribution of the electrodes over the surface of the head and on further improving the usability as well as reducing set-up times in different demanding situations. The measurement electronics will also be miniaturized in order to allow the development of a more streamlined electrode cap.

## Conclusion

In summary, this paper has presented the design and testing of the quick-application six-channel wireless emEEG system. As a result of the good quality EEG signals recorded in various environments and due to its ability to stream the recorded data in real-time, the emEEG system could be employed in prehospital care in the future. The EEG recordings made during ambulance transport would enable the EEG data to be interpreted and to guide the diagnostic process before the patient’s arrival at the hospital.

## Abbreviations

ADC: Analog-to-digital converter; AMS: Altered mental state; CIC: Computer interface card; CMRR: Common-mode rejection ratio; EEG: Electroencephalography; EM: Emergency medicine; emEEG: Emergency EEG; FIR: Finite impulse response; HP: High-pass; IA: Instrumentation amplifier; ICU: Intensive care unit; LP: Low-pass; PMU: Portable measurement unit; SD: Standard deviation; WLAN: Wireless local area network.

## Competing interests

The authors declare that they have no financial and non-financial competing interests that might be perceived to influence the results and/or discussion reported in this article.

## Authors’ contributions

AJ developed the recording software and quick-application cap. He participated in the design of the study, performed the statistical analysis, and drafted the manuscript. AK participated in the design of the study and assisted with the drafting of the manuscript. TS and JV designed and developed the wireless EEG recorder. PK was involved with the design of the system and supervised the whole project. VJ and HH analyzed and interpreted the clinical data. All authors have read and approved the final manuscript.
